# Comparative effects of mineral block, capsule, and tablet supplementation on growth performance, mineral intake, and serum mineral status of thin-tailed Indonesian sheep

**DOI:** 10.14202/vetworld.2026.1287-1299

**Published:** 2026-03-23

**Authors:** Gunawan Gunawan, Erna Winarti, Harwi Kusnadi, Wulandari Wulandari, Ririen Indriawaty Altandjung, Heru Ponco Wardono, Zein Ahmad Baihaqi, Novia Qomariyah, Moh Sofi’ul Anam, Yuni Suranindyah

**Affiliations:** 1 Research Center for Animal Husbandry, National Research and Inovation Agency (BRIN), Jl. Raya Jakarta-Bogor, Cibinong, Bogor 16915, Indonesia; 2 Department of Animal Nutrition and Feed Science, Faculty of Animal Science, Universitas Gadjah Mada, Jl. Fauna, No. 03, Bulaksumur, Yogyakarta 55281, Indonesia; 3 Department of Animal Production, Faculty of Animal Science, Universitas Gadjah Mada, Jl. Fauna, No. 03, Bulaksumur, Yogyakarta 55281, Indonesia

**Keywords:** growth performance, mineral supplementation, serum mineral profile, sheep nutrition, thin-tailed sheep, tropical livestock production

## Abstract

**Background and Aim:**

Optimizing mineral supplementation strategies is crucial to enhance growth performance, mineral utilization, and economic efficiency in small ruminant production systems, especially under tropical smallholder conditions where mineral deficiencies often occur. Different forms of mineral supplements may affect intake behavior, bioavailability, and physiological responses. This study examined the comparative effects of three mineral supplement forms, block, capsule, and tablet, on growth performance, mineral intake, and serum mineral status in thin-tailed Indonesian sheep under tropical management conditions.

**Materials and Methods:**

Forty clinically healthy, thin-tailed Indonesian rams (initial live weight 17.62 ± 3.41 kg; aged 6–8 months) were assigned to a randomized complete block design with four treatments and 10 replicates for a 10-week feeding trial. The treatments included: T0 (control, no mineral supplement), T1 (mineral block), T2 (mineral capsule), and T3 (mineral tablet). All animals received the same basal diet, consisting of cultivated grass (*Pennisetum purpureum*) offered *ad libitum* and wheat pollard concentrate at 1.5% of live weight on a dry matter (DM) basis. Growth performance parameters were analyzed using analysis of covariance with initial body weight as a covariate, while mineral intake and serum mineral concentrations were analyzed using one-way analysis of variance followed by Duncan’s multiple range test. Blood samples were collected on days 0 and 70 for serum mineral analysis.

**Results:**

DM intake did not differ significantly among treatments (p > 0.05). However, mineral supplementation significantly influenced growth performance. Rams receiving mineral blocks (T1) showed the highest average daily gain (71.29 g/head/day), which was 26.6% higher than the control group (56.29 g/head/day) (p < 0.05). Capsule (64.36 g/day) and tablet (65.64 g/day) supplementation produced intermediate responses. Feed gain ratio did not differ among treatments. The highest income over feed cost was recorded in T1 (3,448 IDR/head/day). Mineral supplementation significantly increased calcium and chloride intake compared to the control. Serum potassium and copper concentrations were significantly higher in supplemented groups, whereas calcium, manganese, and zinc levels remained unaffected.

**Conclusion:**

Mineral supplementation improved growth performance, mineral intake, and economic returns in thin-tailed Indonesian sheep without impacting DM intake. Among the tested delivery methods, mineral block supplementation was the most effective, offering the highest growth rate and an economic advantage while maintaining stable serum mineral levels. These results suggest that mineral block supplementation is a practical and scalable approach for enhancing productivity in tropical smallholder sheep production systems.

## INTRODUCTION

Feed availability and quality remain major constraints in smallholder livestock systems in Indonesia. Feeding practices mainly depend on low-quality forages and agricultural by-products that lack essential nutrients, especially protein and energy. Limited access to concentrates and nutritional supplements, mainly because of high costs and insufficient knowledge of balanced ration formulation, often leads to deficiencies in minerals and essential amino acids, which in turn restricts livestock productivity and product quality.

Optimal mineral nutrition is crucial for enhancing growth, nutrient absorption, and overall health in small ruminants. Minerals such as calcium (Ca), phosphorus (P), chlorine (Cl), copper (Cu), and zinc (Zn) are vital for skeletal development, enzyme function, immune response, and metabolic processes [1–4]. However, the success of mineral supplementation relies not only on the amount provided but also on the delivery method. Different forms of supplementation, including mineral blocks, capsules, tablets, and loose premixes, may affect voluntary intake, bioavailability, and physiological responses in ruminants in different ways.

Mineral blocks are widely used because they offer a free-choice delivery system, allowing animals to self-regulate mineral intake based on physiological needs while lowering labor and feed-handling costs [5]. Long-term supplementation with mineral blocks has been shown to enhance serum trace mineral levels, antioxidant status, and immune function in Tibetan sheep [6]. Similarly, mineral supplementation significantly increased serum Ca, P, and Mg levels in Blackbelly sheep during the pre- and postpartum periods under free-grazing conditions in Ecuador, underscoring the importance of maintaining adequate mineral status for reproductive and metabolic health [7].

Despite these advantages, block-based supplementation has certain limitations. Variability in licking behavior among individual animals can lead to uneven mineral intake, potentially causing under- or over-supplementation. Alternatively, controlled delivery systems such as capsules or tablets may offer more precise dosing but could increase labor requirements and handling stress. However, comparative studies assessing the effectiveness of block, capsule, and tablet supplementation forms remain limited, especially for indigenous sheep breeds raised under tropical smallholder systems such as those in Indonesia. Mineral blocks are typically formulated using ingredients such as cement, brewery spent grain, and coffee husks to improve compressive strength and durability, while salt is added to enhance palatability and promote voluntary consumption.

Although mineral requirements of sheep have been extensively studied in temperate production systems, research in tropical conditions remains limited, especially concerning how the physical form of mineral supplementation interacts with the physiological responses of local animals. Environmental factors typical of tropical regions, such as high ambient temperatures, fluctuating humidity, and variable forage quality, can affect feed intake, mineral metabolism, and nutrient utilization efficiency [8]. Therefore, assessing suitable mineral delivery methods in tropical conditions is essential for improving nutritional efficiency and enhancing the resilience of smallholder livestock systems in Indonesia [9, 10].

Blood mineral profiles serve as important indicators of mineral status and help clarify the relationship between mineral intake and animal performance. Adequate serum levels of macro- and microminerals are essential for optimal metabolic function, while deficiencies or imbalances can impair growth, feed efficiency, and physiological processes [11, 12]. Previous studies have demonstrated that mineral source, dosage, and supplementation duration influence growth rate, feed conversion efficiency, and blood biochemical parameters in lambs. For instance, supplementation with organic trace minerals significantly enhanced growth performance and feed conversion efficiency in Zandi lambs compared to non-supplemented controls [13]. However, most previous research has assessed mineral supplementation mainly as a binary intervention (supplemented versus non-supplemented) or by comparing different mineral compositions. Such methods make it difficult to differentiate the effects of mineral composition from those related to the physical form of supplementation.

A previous study in Indonesia demonstrated that supplementation with a herbal mineral block improved serum Ca and P concentrations and enhanced growth performance in Dorper crossbred sheep under restricted feeding and variable forage quality conditions [14]. However, direct comparisons among block, capsule, and tablet forms containing identical mineral formulations remain scarce, particularly in thin-tailed Indonesian sheep. Understanding how different supplementation forms influence mineral intake, serum mineral status, and growth performance under tropical smallholder conditions is essential for developing cost-effective and practical feeding strategies.

Smallholder sheep production systems in the tropics are marked by high temperatures, changing forage quality and mineral levels, limited labor, and irregular access to commercial mineral premixes. These factors pose unique challenges for mineral nutrition management, making the choice of supplementation form just as important as the mineral content itself.

Therefore, this study aimed to evaluate the effects of three mineral supplement delivery forms, block, capsule, and tablet, on growth performance, mineral intake, and serum mineral profiles in thin-tailed Indonesian sheep compared to an unsupplemented control. Specifically, it examined how supplementation affected average daily gain (ADG), feed conversion ratio, mineral consumption, and serum mineral concentrations, as well as the relationship between these factors and growth performance. We hypothesized that mineral block supplementation would yield better performance through improved self-regulation of mineral intake and enhanced mineral utilization, whereas capsule and tablet forms would yield responses intermediate relative to the control.

To the best of our knowledge, this is the first study to systematically compare mineral block, capsule, and tablet supplementation forms using an identical mineral formulation in thin-tailed Indonesian sheep under tropical smallholder feeding conditions. The novelty of this study lies in providing a controlled comparison between *ad libitum* and fixed-dose mineral supplementation strategies while simultaneously evaluating their effects on mineral intake regulation, serum mineral homeostasis, growth performance, and economic returns.

## MATERIALS AND METHODS

### Ethical approval

All experimental procedures involving animals in this study were reviewed and approved by the Animal Ethics Committee of the National Research and Innovation Agency (BRIN), Indonesia, under approval number 1027/KE.02/SK/02/2024. The study was conducted in accordance with internationally accepted guidelines for the care and use of animals in scientific research, ensuring compliance with institutional and national animal welfare regulations.

Before the start of the experiment, all animals underwent clinical health screening to confirm normal physiological condition and suitability for inclusion in the study. Only clinically healthy thin-tailed Indonesian rams with uniform body condition scores and no history of metabolic or infectious disease were enrolled. Animals showing any signs of illness or abnormal physiological status were excluded from the experiment. To standardize parasite status and minimize health-related variability, all rams were administered albendazole (1 mL/12 kg body weight) subcutaneously 1 week before the beginning of the experimental trial.

Throughout the experimental period, animal welfare was carefully monitored by trained personnel under veterinary supervision. The sheep were housed individually in well-ventilated elevated pens designed to maintain hygienic conditions and reduce stress associated with overcrowding. Pens were cleaned daily, and animals had continuous access to clean drinking water and nutritionally adequate feed. Environmental conditions were maintained within the normal range for tropical sheep production systems, and animals were observed daily for behavioral changes, feed intake, and general health status.

All handling procedures, including weighing, feeding, and blood sampling, were carried out using standard livestock management practices to minimize animal discomfort and stress. Blood samples were collected by trained technicians using aseptic techniques and handled according to accepted veterinary clinical procedures to ensure both animal welfare and sample integrity.

The experimental design followed the principles of the 3Rs (Replacement, Reduction, and Refinement) to ensure responsible animal use in research. The number of animals used was limited to the minimum required to obtain statistically reliable results, and all procedures were refined to minimize potential distress or harm to the animals. No invasive surgical procedures or harmful treatments were applied during the study, and no adverse health effects were observed in any of the experimental animals during the trial period.

### Study period and location

The study was conducted from August 2024 to October 2024 at Sembada Farm, located in Selomartani Village, Kalasan Subdistrict, Sleman Regency, Special Region of Yogyakarta, Indonesia (7°43′46.1″ S, 110°26′45.9″ E).

### Experimental animals

A total of 40 clinically healthy thin-tailed Indonesian rams, aged 6-8 months, with an initial live weight of 17.62 ± 3.41 kg and a coefficient of variation of 19.4%, were used in this experiment. Only animals with normal health status, uniform body condition scores, and no history of metabolic disorders were included, whereas those showing any clinical abnormalities were excluded. To standardize parasite status, all rams were administered subcutaneous albendazole (1 mL/12 kg body weight) 1 week before the start of the experiment.

### Experimental design and treatment allocation

The animals were housed individually and assigned to treatments using a randomized complete block design. Before allocation, the rams were stratified into three body weight categories (low, medium, and high) to ensure baseline homogeneity. Initial body weight data were statistically analyzed and found tonot be differ significantly among treatment groups (p > 0.05). Within each body weight category, animals were allocated to pens by lottery to reduce environmental bias and then randomly assigned to one of four dietary treatments, with 10 animals per treatment.

Treatment T0 served as the control and received the current feeding system (CFS) ad libitum, along with a concentrate supplement diet (CSD) offered at 1.5% of live weight on a dry matter (DM) basis. Treatments T1, T2, and T3 received the same basal diet as T0 but were supplemented with minerals in block, capsule, and tablet forms, respectively. The mineral composition was identical across all three supplement forms. Initial body weights were recorded on November 20, 2024, followed by a 14-day adaptation period and a 70-day feeding trial.

### Housing and animal management

The rams were kept in individual elevated pens measuring 1.5 × 1.0 m per animal and equipped with plastic slatted floors to promote sanitation and hygiene. The housing system was open-sided to ensure good natural ventilation. Pens were cleaned daily to reduce manure buildup and lower the risk of disease transmission. During the study, the animals were kept under tropical environmental conditions, with ambient temperatures ranging from 25°C to 28°C and an average relative humidity of about 82%. Animal health and welfare were monitored daily under veterinary supervision in accordance with standard procedures, including regular checks of feed intake, behavior, and physical condition. Any signs of illness were promptly assessed and treated by a veterinarian.

### Basal diet and feeding management

The CFS consisted of cultivated grass (*Pennisetum purpureum*), while the CSD included wheat pollard, fed daily at 1.5% of live weight on a DM basis, as recommended by Lavania [15]. The nutrient and mineral composition of the basal diet was the same for all treatments, and only the form of mineral supplementation varied among groups. Details of the dietary treatments and the nutrient and mineral composition of feed ingredients are shown in Tables 1, 2, and 3.

**Table 1:** Dietary treatments of the experimental diet.

Treatments	Composition of the feed
T0	CFS *ad libitum* + CSD offered at 1.5 % of LW/day on a DM basis
T1	CFS *ad libitum* + CSD offered at 1.5 % of LW/day on a DM basis + mineral supplement (block)
T2	CFS *ad libitum* + CSD offered at 1.5 % of LW/day on a DM basis + mineral supplement (capsule)
T3	CFS *ad libitum* + CSD offered at 1.5 % of LW/day on a DM basis + mineral supplementation (tablet)

Note: CFS = current feeding system, consisting of cultivated grass (*Pennisetum purpureum*); CSD = concentrate supplement diet, which includes wheat pollard; LW = live weight; DM = dry matter.

**Table 2:** Feed nutrient content of *Pennisetum purpureum* and wheat pollard.

Ingredient	DM (% as fed)	Ash (%DM)	CP (%DM)	EE (%DM)	CF (%DM)	NFE (%DM)	TDN (%DM)	Price (IDR/ kg DM)
*Pennisetum purpureum*	34.20	11.23	7.42	0.97	34.73	45.56	57.18	1462
Wheat pollard	91.23	4.66	16.16	3.79	9.06	66.33	72.67	4494

Note: DM = dry matter, CP = crude protein, EE = ether extract, CF = crude fiber, NFE = nitrogen-free extract, TDN = total digestible nutrient, were calculated as described by Harris *et al.* [1]: TDN_1_ = –54.572 + 6.769 (CF) + 51.083 (EE) + 1.851 (NFE) + 0.334 (CP) + 0.049(CF)² + 3.384 (EE)² + 0.086 (CF)(NFE) + 0.687 (EE)(NFE) + 0.942 (EE)(CP) + 0.112 (EE)² (CP); TDN_2_ = –202.686–1.357 × (CF) + 2.638 × (EE) + 3.003 × (NFE) + 2.347 × (CP) + 0.046 × (CF)² + 0.647 × (EE)² + 0.041 × (CF)(NFE) – 0.081(EE)(NFE) + 0.553(EE)(CP) – 0.046(EE)² × (CP). The price of the feed ingredient is calculated from the market price in this region, i.e., the price of *Pennisetum purpureum* = 500 IDR/kg as fed and wheat pollard = 4100 IDR/kg as fed. Indonesian Rupiah (IDR).

**Table 3:** Mineral content of feed ingredients.

Ingredient	Ca (%)	P (%)	K (%)	Cl (%)	Fe (mg/kg)	Mn (mg/kg)	Cu (mg/kg)	Zn (mg/kg)
*Pennisetum purpureum*	2.68	0.39	8.20	0.51	4560.0	365.9	55.9	275.3
Wheat pollard	0.89	0.67	3.72	0.13	1540.0	1320.0	110.2	576.1
Mineral supplement*	25.44	0.21	0.44	10.84	1860.0	323.7	23.9	77.5

Note:

* Mineral supplements: a composition of 40% salt, 26% white cement, 20% minerals, 9% beer dregs, and 5% coffee husk in the form of mineral blocks, capsules, and tablets; Ca = calcium, P = phosphorus, K = potassium, Cl = chlorine, Fe = iron, Mn = manganese, Cu = copper, Zn = zinc.

The animals underwent a two-week adaptation period, during which the amount of CSD was gradually increased until it reached 1.5% of live weight per day. The daily CSD allowance was split into two equal meals, offered in the morning and afternoon. Cultivated grass (*P. purpureum*) was provided freely throughout the day, with fresh forage given twice daily to maintain quality. The forage provided exceeded voluntary intake, resulting in about 10% refusals over 24 h, which served as a management indicator rather than a feed restriction. Additional forage remained available in the trough between feedings. No refusals of the CSD were observed.

Feed was offered twice daily at 08:00 and 16:00, with concentrate supplied before forage to encourage more uniform nutrient intake. Feed and drinking water were available ad libitum throughout the experiment. No mineral analysis of drinking water was performed. Based on live weight recorded every 2 weeks, the amount of CSD offered to each animal was adjusted accordingly, and daily individual rations were prepared. DM analysis of CSD samples was conducted at the same intervals.

### Preparation and administration of mineral supplements

Mineral supplements were prepared weekly by the research team with help from trained animal caretakers. Each weekly batch was designed to cover 7 days and then divided into daily doses to maintain consistency, freshness, and accurate dosing throughout the study.

In T1, mineral supplements were provided as 500 g mineral blocks suspended in each pen, allowing free-choice intake based on individual needs. In T2, the supplements were given as capsules, with each ram receiving three capsules once daily, each containing 1 g of minerals, for a total daily dose of 3 g. In T3, the supplements were supplied as tablets, with each ram receiving one tablet daily containing 3 g of minerals, matching the total daily dose in T2. Therefore, mineral intake was consistent across T2 and T3, and any differences between treatments were due to the supplement form rather than the dose. All capsules and tablets were fully consumed by the animals.

The mineral supplements used in T1, T2, and T3 consisted of 40% salt, 26% white cement, 20% minerals, 9% brewer’s grains, and 5% coffee husk. Cement was added as a binder to enhance the hardness and water resistance of the mineral block. Despite the relatively high inclusion level, actual cement intake remained low because voluntary block consumption was minimal under free-choice feeding conditions. Previous studies on molasses-urea and mineral block technologies have shown that cement-based binders can increase block durability without negative effects when their contribution to total dietary DM remains small [16, 17].

For block preparation, all dry ingredients except white cement were first thoroughly mixed. White cement was added afterwards once the other ingredients were evenly blended. Water was then gradually added until a uniform mixture was achieved. The mixture was poured into polyvinyl chloride pipe molds and manually compacted to ensure sufficient density. The blocks were then air-dried at ambient temperature for several days until fully hardened. Hardness and texture were evaluated visually and by touch to confirm adequate solidity for licking without excessive crumbling. Capsules and tablets were placed directly into the feed troughs of individual pens to ensure full intake and ease of compliance monitoring. Mineral blocks were suspended above the feed troughs to prevent contamination and access by animals in neighboring pens. The elevated pens, covered with asbestos roofing, protected the blocks from rain and environmental contamination. The duration of licking activity was not recorded during the trial.

### Measurement of feed and mineral intake

Trained technicians recorded the daily intake of cultivated grass (*P. purpureum*) and wheat pollard using digital scales with a 50 kg capacity. The amounts of feed offered and refused were measured and logged daily by the research team. Feed refusals were collected and weighed each morning before the first feeding. Representative refusal samples were oven-dried at 55°C to determine their DM content. Feed allowances were calculated based on individual body weight and provided in quantities exceeding estimated nutritional needs to prevent restriction, as indicated by the presence of refusals. Feed intake and nutrient consumption were expressed on a DM basis.

Mineral supplement intake was evaluated every two weeks. Consumption of mineral blocks was estimated by the difference in block weight between two consecutive measurements. Intake of capsule and tablet supplements was similarly calculated by subtracting the remaining supplement weight from the initial amount provided. All mineral supplement intake values were expressed as grams per head per day on a DM basis.

### Growth performance evaluation

Live weight was measured every 2 weeks before morning feeding using digital scales. ADG was calculated as: ADG = (final live weight − initial live weight) / 70 days.

Feed gain ratio (FGR) was calculated as: FGR = DM intake / ADG.

Income over feed cost (IOFC) was calculated as: IOFC = (ADG × market price in IDR per kg of live weight, based on prices from local farmers [approximately Rp75,000/kg]) − daily feed cost.

### Chemical and mineral analysis of feeds and supplements

Feed and supplement samples were dried at 55°C, blended, and sieved through a 1 mm mesh before analysis. Proximate composition, including crude protein (AOAC 978.04), ether extract (AOAC 930.09), crude fiber (AOAC 930.10), and nitrogen-free extract, was determined in triplicate at the Biochemistry of Nutrition Laboratory, Faculty of Animal Science, Universitas Gadjah Mada, Indonesia, following official AOAC procedures.

Mineral concentrations of Ca, phosphorus, potassium (K), Cl, iron, manganese (Mn), Cu, and Zn were determined using X-ray fluorescence at the National Research and Innovation Agency facilities. Analyses were performed with a Thermo Scientific Niton XL3t 500 handheld analyzer. Samples were oven-dried at 60°C for 24 h, ground to <80 mesh (177 μm) using a stainless steel mill, and analyzed in triplicate with three readings per sample (60 s/spot, three spots per sample). Calibration was conducted using NIST SRM 1570a spinach leaves and in-house feed certified reference materials. The limits of detection were 15 ppm for Ca, 20 ppm for phosphorus, and 10 ppm for magnesium. The method achieved a relative standard deviation of 2.1% (n = 10 replicates) and spike recoveries ranged from 96% to 103%.

### Blood sampling and serum mineral analysis

Serum samples were collected from three randomly chosen rams per treatment group as a representative subset of the experimental population. Blood samples were taken in the morning before feeding to reduce postprandial variation. Samples were collected into plain and ethylenediaminetetraacetic acid tubes, and serum was separated from the plain tubes after centrifugation [18]. All blood and serum samples were transported to the laboratory in a cooling box with ice packs at approximately 4°C and processed within 1 h to maintain sample integrity. Serum mineral analysis was performed to provide additional physiological information on mineral status in response to the dietary treatments, while the primary outcomes of the experiment remained growth performance and mineral intake measured in all animals.

Serum mineral concentrations were measured using an Agilent 280FS Atomic Absorption Spectropho-tometer in air-acetylene flame mode, with wavelengths set at 766.5 nm for K, 422.7 nm for Ca, and 285.2 nm for magnesium, following the manufacturer’s instructions. Calibration standards were prepared from Titrisol® CRM Merck (Cat. No. 170230; 1000 mg/L K, traceable to NIST SRM). Quality control included ultra-pure aquades blanks (<0.001 mg/L per batch), duplicate analysis of 15% of the total samples (CV = 3.2%), and recovery tests after adding a 2 mg/L standard to a serum pool, resulting in recoveries of 98.5% for K, 101.0% for Ca, 97.8% for magnesium, and 99.5% for Zn. Changes in serum mineral levels were determined by subtracting day 0 values from day 0 values. The use of only three animals per treatment for serum analysis was due to logistical constraints and is acknowledged as a limitation of the study. Quality assurance was further supported by calibration with known standards, spike-recovery testing, and calculating the limit of detection and quantification based on blank measurements and signal standard deviations.

### Statistical analysis

The experiment was carried out using a randomized complete block design with four dietary treatments and 10 animals in each treatment group. Blocking was based on initial live weight to minimize baseline variability before randomly assigning the subjects. Growth performance variables were measured in all animals and analyzed using analysis of covariance, with initial live weight included as a covariate to control for baseline differences that could affect growth responses [19]. When treatment effects were significant, means were compared using Duncan’s multiple range test [20].

Mineral intake and changes in serum mineral levels were analyzed using one-way analysis of variance, followed by Duncan’s multiple range test for pairwise comparisons. Assumptions of normality and homogeneity of variance were tested with the Shapiro-Wilk and Levene’s tests, respectively, and all assumptions were met before conducting the analyses. Statistical analysis was carried out using R software version 4.3.1, and differences were deemed significant at p < 0.05.

### Blinding statement

Blinding was not feasible due to the nature of the dietary treatments. The technician responsible for feed preparation and measurements was aware of treatment allocation; however, objective measurement methods and standardized protocols were used throughout the study to minimize observer bias.

## RESULTS

### Growth performance

DM intake (DMI) did not significantly differ between rams in the control group without mineral supplementation (T0) and those receiving mineral supplementation (T1, T2, and T3) (p > 0.05; Table 4). Likewise, when DMI was expressed as a percentage of live weight, no significant differences were observed among treatments.

**Table 4:** Growth performance of rams under control (T0) without mineral supplementation and with mineral supplementation (T1, T2, and T3) for 10 weeks.

Variables	T0	T1	T2	T3
DMI (g/day) ^ns^	703.22 ± 7.86	707.36 ± 10.12	709.40 ± 6.96	703.99 ± 8.07
DMI (% LW) ^ns^	4.09 ± 0.19	4.17 ± 0.25	4.19 ± 0.29	4.12 ± 0.24
Initial LW (kg) ^ns^	17.63 ± 1.09	17.62 ± 1.22	17.62 ± 1.19	17.63 ± 1.03
ADG (g/head/day)*	56.29 ± 4.22^b^	71.29 ± 5.05^a^	64.36 ± 5.34^ab^	65.64 ± 5.95^ab^
FGR (DMI/ADG) ^ns^	13.08 ± 0.93	10.39 ± 0.75	11.76 ± 1.03	11.68 ± 1.21
IOFC (IDR/head/day)*	2346 ± 306^b^	3448 ± 370^a^	2591 ± 411^ab^	3036 ± 453^ab^

Note: Values are expressed as mean ± standard error of the mean (n = 10). DMI = dry matter intake, LW = live weight, ADG = average daily gain, FGR = feed:gain ratio, IOFC = income over feed cost, IDR = Indonesian Rupiah, ns = not significant (no superscript).

* Different superscripts within a row indicate significant differences (*p* < 0.05). T0 = control (no mineral supplement), T1 = mineral block, T2 = mineral capsule, T3 = mineral tablet.

Mineral supplementation affected growth performance, especially the ADG. Rams given mineral blocks (T1) showed a significantly higher ADG (71.29 g/day) than those in the control group (T0; 56.29 g/day) (p < 0.05), which is about a 26.6% increase. Rams receiving mineral supplements in capsule (T2; 64.36 g/day) and tablet (T3; 65.64 g/day) forms had intermediate ADG values that did not differ significantly from either the control or the mineral block group.

The FGR was not significantly influenced by the treatments. However, IOFC varied significantly among groups. Rams given mineral block supplementation (T1) achieved the highest IOFC (3,448 IDR/head/day), which was significantly higher than the control group (2,346 IDR/head/day) (*p* < 0.05). Conversely, IOFC values for the capsule (T2; 2,591 IDR/head/day) and tablet (T3; 3,036 IDR/head/day) treatments did not differ significantly from those of either the control or mineral block groups (Table 4).

### Mineral consumption

Total mineral intake varied significantly between the supplemented and non-supplemented groups. Rams receiving mineral supplementation (T1, T2, and T3) showed significantly higher total mineral consumption than those in the control group (T0) (p < 0.05; Table 5). This increase was especially noticeable for Ca and chloride (Cl) intake.

**Table 5:** Mineral intake (g/head per day) in control (T0) and treatment groups (T1, T2, and T3).

Mineral intake	T0	T1	T2	T3
Calcium*	13.847 ± 0.075^b^	14.532 ± 0.125^a^	14.582 ± 0.060^a^	14.643 ± 0.079^a^
Phosphorus ^ns^	3.525 ± 0.052	3.541 ± 0.067	3.553 ± 0.047	3.519 ± 0.052
Potassium ^ns^	4.515 ± 0.030	4.520 ± 0.038	4.530 ± 0.025	4.500 ± 0.033
Chlorine *	2.525 ± 0.011^c^	2.815 ± 0.031^b^	2.829 ± 0.009^ab^	2.877 ± 0.012^a^
Iron ^ns^	0.236 ± 0.001	0.237 ± 0.002	0.237 ± 0.001	0.236 ± 0.002
Manganese ^ns^	0.052 ± 0.001	0.053 ± 0.001	0.053 ± 0.001	0.052 ± 0.001
Copper ^ns^	0.005 ± 0.000	0.005 ± 0.000	0.005 ± 0.000	0.005 ± 0.000
Zinc ^ns^	0.028 ± 0.000	0.028 ± 0.001	0.028 ± 0.000	0.028 ± 0.001

Note: Values are expressed as mean ± standard error of the mean (n = 10).

* Different superscripts within a row indicate significant differences among treatments (p < 0.05). ns = not significant (no superscript). T0 = control (no mineral supplement), T1 = mineral block, T2 = mineral capsule, T3 = mineral tablet.

In the supplemented treatments, mineral intake came from cultivated grass, wheat pollard, and the respective mineral supplements, while in the control group, it was only from grass and wheat pollard. Despite these differences compared to the control group, there were no significant differences in total mineral intake among the three supplementation groups (T1, T2, and T3) (p > 0.05). Variability in mineral intake among individual animals was observed, particularly in the mineral block group (T1), likely related to differences in licking behavior among rams.

### Serum mineral profiles

Changes in serum mineral levels after the 70-day feeding period are shown in Table 6. These changes were calculated by subtracting the baseline (day 0) levels from the values at the end of the study (day 70).

**Table 6:** Changes in serum mineral concentrations (mg/dL) in rams over a 70-day experimental period in the control (T0) and treatment (T1, T2, and T3) groups.

Serum Mineral	T0	T1	T2	T3
Calcium ^ns^	74.87±0.54	75.30±0.42	79.87±2.02	75.77±4.86
Potassium*	273.80±4.26^d^	293.50±0.58^c^	309.33±2.96^b^	348.40±2.54^a^
Manganese ^ns^	0.15±0.07	0.15±0.05	0.19±0.09	0.16±0.04
Copper *	0.04±0.01^b^	0.11±0.03^ab^	0.13±0.02^a^	0.10±0.03^ab^
Zinc ^ns^	0.39±0.09	0.55±0.09	0.54±0.13	0.46±0.03

Note: Values are expressed as mean ± standard error of the mean (n = 3).

* Different superscripts within a row indicate significant differences among treatments (*p* < 0.05). ns = not significant (*p* > 0.05). T0 = control (no mineral supplement), T1 = mineral block, T2 = mineral capsule, T3 = mineral tablet. Changes in serum mineral concentrations were calculated as the difference between the values at the end of the experiment (day 70) and those at baseline (day 0) before the feeding trial.

Mineral supplementation notably affected specific serum mineral levels. K and Cu concentrations were significantly higher in the supplemented groups compared to the control group (p < 0.05). The highest serum K level was seen in the tablet-supplemented group (T3; 348.40 ± 2.54 mg/dL), followed by the capsule (T2) and block (T1) treatment groups, while the lowest value was recorded in the control group (273.80 ± 4.26 mg/dL).

Similarly, serum Cu concentrations were higher in all supplemented groups (T1–T3) compared to the control group (T0), with the largest increase seen in the capsule treatment (T2; 0.13 ± 0.04 mg/dL). In contrast, serum levels of Ca, Mn, and Zn were not significantly affected by mineral supplementation (p > 0.05). These results suggest that supplementation selectively affected certain serum minerals rather than causing a uniform change across all mineral parameters.

## DISCUSSION

### Effect of mineral delivery form on physiological responses

The current findings suggest that the physical form of mineral supplementation significantly influences physiological responses in thin-tailed sheep, rather than just the total amount of minerals consumed. Mineral blocks enable animals to control their intake voluntarily based on their physiological needs, while capsule and tablet supplements provide a fixed-dose. This self-regulated intake likely supports a gradual mineral release in the rumen, helping to reduce short-term fluctuations in mineral availability and enhance systemic mineral balance.

### Effects of mineral block supplementation on growth performance

Although mineral block supplementation has been widely studied in temperate livestock systems, its effectiveness under tropical management conditions remains less understood. The current findings show that mineral block supplementation can significantly improve the growth performance of sheep raised in tropical feeding environments, where mineral deficiencies in forage-based diets are common.

Rams receiving mineral block supplementation (T1) showed significantly greater growth performance, FGR, and IOFC compared to the control group (T0). The higher ADG and improved economic returns observed in T1 suggest better nutrient utilization and metabolic efficiency. This improvement may be due to the balanced supply of macro- and microminerals that promote rumen fermentation and enzymatic activity. Enhanced microbial metabolism increases the production of volatile fatty acids and microbial protein, which are then converted into body tissue [21–23].

Adequate mineral availability also stimulates microbial enzyme activity, enhances feed digestibility, and promotes protein synthesis [24–26]. Similar improvements in nutrient utilization and productivity following mineral block supplementation have been reported in small ruminants by Castro *et al*. [27], Gooneratne *et al*. [28], and Mobashar *et al*. [29]. In the present study, the improved ADG and IOFC associated with mineral block supplementation support previous findings highlighting the importance of adequate mineral supply for metabolic efficiency and growth in small ruminants [30–32].

The licking behavior observed in rams receiving mineral blocks allows animals to regulate mineral intake based on physiological demand, thereby reducing the risk of both deficiency and excessive intake [33–35]. Additionally, the gradual release of minerals from blocks prevents short-term spikes in concentration that can occur with bolus dosing through capsules or tablets, potentially enhancing absorption efficiency and lowering metabolic stress [26]. These findings indicate that steady mineral availability aligned with rumen activity may be more advantageous than precise pulse dosing for grazing sheep.

### Economic implications of mineral supplementation

From an economic perspective, moderate fluctuations in mineral supplement prices are unlikely to significantly alter the ranking of IOFC among treatments, as feed costs account for the largest share of production expenses. However, sharp increases in mineral block prices could decrease the economic benefit of this supplementation strategy. Based on the price assumptions used in this study, mineral block supplementation resulted in higher IOFC than conventional methods. This benefit is expected to continue under relatively stable prices, although it could diminish if mineral block prices increase substantially relative to other supplements.

The lack of notable differences in FGR among treatments aligns with the similar DMI values observed across groups.

### Mineral intake and nutritional balance

Ca intake was significantly higher in all mineral-supplemented groups (T1–T3) than in the control group (T0), whereas no significant differences were observed among the supplemented treatments. This suggests that mineral supplementation alone, rather than the specific form used, was enough to boost Ca intake. The similar Ca consumption across supplemented groups indicates that physiological homeostatic mechanisms kept intake stable once dietary needs were met [23, 36, 37].

Ca plays a vital role in energy metabolism, skeletal development, and muscle contraction [4, 22, 38, 39]. Increased Ca absorption and use have been linked to better growth performance in small ruminants [40]. However, the stable serum Ca levels seen in this study suggest that circulating Ca concentrations are tightly controlled through endocrine mechanisms involving parathyroid hormone and calcitonin [41–43].

The observed Ca intake in supplemented groups (14.5–14.6 g/day) exceeded the National Research Council maintenance requirements for growing sheep [44], indicating that the mineral formulation provided an adequate safety margin to prevent deficiency. Although mineral block consumption showed greater variability between individuals compared with capsule and tablet supplementation, the total mineral intake remained higher than that of the control group and contributed to improved growth performance.

### Patterns of Cl and trace mineral intake

Cl intake varied slightly among supplementation forms, with the highest value observed in the tablet treatment, followed by the capsule and mineral block treatments. This pattern may reflect differences in intake behavior related to controlled-dose supplements compared to free-choice mineral blocks.

Overall, mineral consumption patterns showed that most macro- and micro-mineral intakes exceeded the physiological requirements of rams weighing 17–22 kg, as defined by McDowell *et al*. [41], NRC [44], and Kaneko *et al*. [18]. In contrast, K and Cu intake did not differ significantly among treatments, indicating that the basal diet combined with endogenous regulatory mechanisms was enough to maintain mineral balance.

### Effects of supplementation on serum mineral profiles

Among the serum minerals analyzed, only K and Cu showed significant differences among treatments. The higher serum Cu concentrations in the supplemented groups suggest improved Cu availability and absorption systemically after mineral supplementation. The highest serum Cu level was observed in the capsule treatment, while the mineral block and tablet treatments had intermediate values. This pattern indicates that the form and method of mineral delivery can affect Cu bioavailability regardless of total intake.

Cu plays a vital role in redox regulation, hemoglobin synthesis, and mitochondrial metabolism [21, 45–47]. Keeping Cu within its normal range is crucial for metabolic efficiency while reducing antagonistic interactions with other trace minerals such as Zn and Fe [48].

### Role of K in metabolic regulation

Serum K concentrations increased gradually from the control group to the tablet treatment. K plays a key role in osmotic regulation, nerve transmission, and muscle contraction, and adequate K levels support optimal rumen motility and digestion [49]. The moderate increase in serum K seen in supplemented groups may help improve nutrient transport and metabolic efficiency [23, 26].

### Mineral homeostasis and physiological safety

The observed serum mineral levels show that mineral supplementation kept mineral status within normal ranges for sheep [50]. Serum Ca and K levels in the supplemented groups were well above deficiency thresholds, while values in the control group were nearer to the lower boundary of adequacy, indicating a possible risk of subclinical deficiency under typical feeding conditions. Importantly, no signs of mineral toxicity appeared in any treatment group, confirming the safety of the supplementation approaches used.

The consistent serum levels of Ca, Mn, and Zn across different treatments further demonstrate effective physiological regulation of mineral balance. Similar results have been reported by Hassan *et al*. [51] and Ramírez-Bribiesca *et al*. [5], who found that mineral block supplementation helps maintain stable mineral levels and boosts productivity in tropical conditions.

### Implications for tropical smallholder livestock systems

Collectively, these findings indicate that mineral block supplementation not only boosts mineral intake but also enhances rumen microbial efficiency and overall metabolic stability. The slow release of minerals from blocks through licking likely helps maintain ruminal osmolarity and microbial enzyme activity, which in turn improves the use of essential cofactors like Ca, Zn, and Cu involved in oxidative and anabolic metabolic processes.

Such supplementation systems offer a practical and scalable approach for boosting productivity in tropical smallholder livestock systems, where variability in feed quality and labor limitations often hinder animal performance.

### Comparison with capsule and tablet supplementation

The lack of clear superiority between capsule and tablet supplementation indicates that precise dosing alone does not necessarily lead to better biological efficiency. Although these delivery methods provide controlled mineral intake, they may ignore behavioral factors of consumption and ruminal adaptation processes. Conversely, self-regulated intake with mineral blocks allows for gradual physiological adjustment and may therefore better enhance mineral utilization in smallholder settings.

### Study limitations and future research directions

Several limitations should be recognized. Nutrient digestibility was not directly assessed, which restricts interpretation of the mechanisms behind improved nutrient utilization. Additionally, serum mineral levels were measured in a small subset of animals (n = 3 per treatment), and the study duration was relatively short at 10 weeks.

Future research should include larger sample sizes for blood biochemical analyses, direct measurements of nutrient digestibility, and longer experimental durations. Additional studies could also explore the effects of mineral supplementation on reproductive performance, immune responses, and rumen microbial ecology in tropical production systems.

### Strengths and scientific contributions of the study

A major strength of this study is the integration of physiological and economic outcomes within a single analytical framework. Growth performance was evaluated using analysis of covariance to control for initial body weight differences. Mineral intake was quantified, and systemic mineral regulation was assessed through serum Ca, Mn, and Zn profiles. These biological responses were further linked to IOFC, providing a direct measure of financial return.

This comprehensive physiological and economic assessment shows that mineral block supplementation can improve both biological efficiency and economic profitability, helping to connect research in animal nutrition with practical decision-making for smallholder farmers.

## CONCLUSION

This study showed that the method of mineral supplementation significantly affects the productivity and mineral status of thin-tailed Indonesian sheep raised under tropical smallholder feeding conditions. Mineral block supplementation (T1) led to the highest growth performance, with a 26.6% increase in ADG compared to the control group, while DM intake and FGR remained unchanged. Mineral-supplemented groups also had higher mineral intake, especially Ca and Cl, compared to the control group. Additionally, serum K and Cu levels increased significantly after supplementation, while Ca, Mn, and Zn stayed stable, indicating effective regulation of mineral balance. Among the delivery systems tested, mineral blocks offered the highest economic return, as shown by the greatest income over feed costs.

From a practical standpoint, mineral block supplementation offers a feasible and labor-efficient way to enhance sheep productivity in tropical smallholder systems. The free-choice intake of mineral blocks enables animals to self-regulate their mineral consumption based on their physiological needs, providing a steady and gradual supply of essential minerals that support rumen microbial activity, nutrient absorption, and metabolic balance. This method also lowers handling stress linked to forced dosing and presents a cost-effective approach that smallholder farmers with limited access to commercial feed supplements can easily adopt.

Overall, the findings emphasize that the effectiveness of mineral supplementation depends not only on mineral composition but also on the delivery system. Mineral block supplementation offers a balanced combination of biological efficiency, economic benefits, and practical applicability under tropical production conditions. Therefore, adopting mineral block technology may help improve sheep productivity, nutritional management, and economic sustainability in smallholder livestock systems.

## DATA AVAILABILITY

The datasets generated and analyzed in this study are available from the corresponding author upon reasonable request.

## AUTHORS’ CONTRIBUTIONS

GG: Designed the study, interpreted, analyzed, and validated the data, and drafted the manuscript. EW, HK, W, RIA, and HPW: Data collection and statistical analysis. ZAB and NQ: Data validation and manuscript preparation. MSA and YS: Manuscript preparation and critical evaluation. All authors have read and approved the final version of the manuscript.
